# A phase I study with MAG-camptothecin intravenously administered weekly for 3 weeks in a 4-week cycle in adult patients with solid tumours

**DOI:** 10.1038/sj.bjc.6601811

**Published:** 2004-05-18

**Authors:** F M Wachters, H J M Groen, J G Maring, J A Gietema, M Porro, H Dumez, E G E de Vries, A T van Oosterom

**Affiliations:** 1 Department of Pulmonary Diseases, University Hospital Groningen, PO Box 30.001, 9700 RB Groningen, The Netherlands; 2 Diaconessenhuis Meppel, PO Box 502, 7940 AM Meppel, The Netherlands; 3 Department of Medical Oncology, University Hospital Groningen, PO Box 30.001, 9700 RB Groningen, The Netherlands; 4 Pfizer, Viale Pasteur 10, 20014 Nerviano (Milan), Italy; 5 Department of Medical Oncology, University Hospital Gasthuisberg, Herestraat 49, 3000 Leuven, Belgium

**Keywords:** camptothecin, clinical trials, phase I, drug carriers, drug delivery systems, pharmacokinetics, polymers

## Abstract

In MAG-camptothecin (MAG-CPT), the topoisomerase inhibitor camptothecin is linked to a water-soluble polymer. Preclinical experiments showed enhanced antitumour efficacy and limited toxicity compared to camptothecin alone. Prior phase I trials guided the regimen used in this study. The objectives were to determine the maximum tolerated dose, dose-limiting toxicities, safety profile, and pharmacokinetics of weekly MAG-CPT. Patients with solid tumours received MAG-CPT intravenously administered weekly for 3 weeks in 4-week cycles. At the starting dose level (80 mg m^−2^ week^−1^), no dose-limiting toxicities occurred during the first cycle (*n*=3). Subsequently, three patients were enrolled at the second dose level (120 mg m^−2^ week^−1^). Two of three patients at the 80 mg m^−2^ week^−1^ cohort developed haemorrhagic cystitis (grade 1/3 dysuria and grade 2/3 haematuria) during the second and third cycles. Next, the 80 mg m^−2^ week^−1^ cohort was enlarged to a total of six patients. One other patient at this dose level experienced grade 1 haematuria. At 120 mg m^−2^ week^−1^, grade 1 bladder toxicity occurred in two of three patients. Dose escalation was stopped at 120 mg m^−2^ week^−1^. Cumulative bladder toxicity was dose-limiting toxicity at 80 mg m^−2^ week^−1^. Pharmacokinetics revealed highly variable urinary camptothecin excretion, associated with bladder toxicity. Due to cumulative bladder toxicity, weekly MAG-CPT is not a suitable regimen for treatment of patients with solid tumours.

Camptothecins (CPTs) are a maturing class of anticancer agents with significant clinical activity against a broad range of malignancies (Kim and Lim, 2002). As the topoisomerase I inhibitor CPT is extremely insoluble, the first phase I trials in the early 1970s were performed with the less active water-soluble carboxylate salt of CPT (Gottlieb *et al*, 1970; Muggia *et al*, 1972). Although some antitumour activity was observed, severe haematologic toxicity and haemorrhagic cystitis hampered the first development. The observation that an acidic environment (e.g. urine) favours the formation of the insoluble CPT lactone suggested an explanation for the prolonged bladder toxicity observed with sodium CPT, as well as a potential approach for alleviating this toxic effect by alkalisation of the urine.

In MAG-camptothecin (MAG-CPT, PNU-166148), CPT is covalently linked to a water-soluble polymer (MAG). MAG-CPT is a copolymer of *N*-(hydroxypropyl)methacrylamide, (20-*O*-(*N*-methacryloyl-glycyl-aminohexanoyl-glycyl)camptothecin) and *N*-(2-hydroxypropyl) methacryloyl-glycinamide. Binding of polymers to cytotoxic drugs may enhance the therapeutic activity and reduce toxicity mainly by altering their pharmacokinetics and biodistribution (Kim and Lim, 2002). Via the so-called enhanced permeability and retention effect, polymeric drug conjugates facilitate the solid tumour targeting of chemotherapeutic agents, in both animal models and man (Maeda and Matsumura, 1989; Duncan, 1999). This enhanced permeability and retention effect can be attributed to leaky tumour vessels allowing macromolecular extravasation not occurring in normal tissues and lack of effective tumour lymphatic drainage preventing clearance of the penetrant macromolecules and promoting their accumulation (Duncan, 1999). The enhanced permeability and retention effect has been observed in many experimental and human solid tumours (Maeda *et al*, 2000).

Experiments in healthy mice showed about five-fold lower plasma levels of MAG-CPT compared to the highest tolerated dose of CPT administered in classical vehicles (Caiolfa *et al*, 2000). Whole-body autoradiographs in HT29 human colon carcinoma-bearing mice demonstrated evident tumour radioactivity uptake after intravenous injection of MAG-(^3^H)CPT, but not after injection of (^3^H)CPT (Caiolfa *et al*, 2000). MAG-CPT was better tolerated and tumour inhibition was observed at similar and even lower doses compared to CPT alone (Caiolfa *et al*, 2000).

Recently, two phase I studies with MAG-CPT have been performed. The first study indicated that a single dose of 200 mg m^−2^ MAG-CPT in a 4-week schedule caused no toxicity (De Bono *et al*, 2000), while at 240 mg m^−2^ three dose-limiting toxicities (DLTs) (neutropenic sepsis, grade 4 thrombocytopenia and grade 3 diarrhoea) occurred. In the second study, patients with solid tumours received MAG-CPT administrated over 3 consecutive days every 4 weeks (Schoemaker *et al*, 2002). Dose-limiting toxicity was cumulative bladder toxicity, occurring in two out of three patients treated at a dose of 85 mg m^−2^ day^−1^. Pharmacokinetic analysis in both studies showed that carrier-bound CPT has linear kinetics. The area under the curves (AUCs) following administration of MAG-CPT as a single dose or administration over three consecutive days are comparable. However, the maximum plasma concentration (*C*_max_) levels of carrier-bound and released CPT were three to five times lower when MAG-CPT was administered over three consecutive days, compared to a single dose administration. Therefore, when exposure to CPT is similar for both administrations, a lower *C*_max_ might give less toxicity. However, as a result of the relatively long half-life of both bound and free CPT, accumulation in plasma was observed during the 3 days of administration. This accumulation might be the explanation for the dose-related toxicity observed in this trial, suggesting that toxicity can be avoided by increasing the time between dosing (Schoemaker *et al*, 2002). This answer was sought in the present study.

In this phase I trial, MAG-CPT was administered weekly for 3 weeks in a 4-week cycle in adult patients with refractory or resistant solid tumours. The primary objectives were to determine the maximum tolerated dose and DLTs, and to define the safety profile of MAG-CPT. The secondary objective was the evaluation of pharmacokinetics. Based on the results of the above-mentioned phase I studies, a starting dose of 80 mg m^−2^ week^−1^ was chosen (total dose 240 mg m^−2^ cycle^−1^).

## PATIENTS AND METHODS

### Patient selection

The study was performed in Leuven, Belgium and Groningen, The Netherlands. Patients were included if they had histological or cytological confirmed malignant tumours for whom no recognised therapy was available. Patients may have received prior chemo-, hormone-, or immunotherapy. Patients must have completed any prior chemotherapy 4 weeks before study entry. Previous treatment with CPTs (e.g. topotecan, irinotecan, 9-AC) was not allowed. Prior radiotherapy was allowed as long as no more than 25% of the bone marrow was irradiated. Patients had to be recovered from all acute toxic effects from any prior therapy, excluding alopecia and Common Toxicity Criteria (CTC) grade 1 neurotoxicity residual from prior chemotherapy. Patients (⩾18 years) had to have a performance status ⩽2 according to the Eastern Cooperative Oncology Group (ECOG) scale, and a life expectancy of at least 12 weeks. Adequate organ functions were required such as bone marrow function (neutrophils ⩾1.5 × 10^9^ l^−1^ and platelets ⩾100 × 10^9^ l^−1^), renal function (serum creatinine <133 *μ*mol l^−1^) and liver function, defined as total bilirubin, serum alanine aminotransferase (ALAT) and serum aspartate aminotransferase (ASAT) within the normal range, or for patients with liver metastases serum ALAT and ASAT less than five times the upper limit of normal and total bilirubin less than 1.5 times the upper limit of normal. Patients with haematologic malignancies, known brain or leptomeningeal disease, more than three prior chemotherapy regimens, previous high-dose chemotherapy requiring (autologous) bone marrow transplantation or peripheral stem cell reconstitution, and known hepatitis B or HIV positive or AIDS-related illness were excluded. Pregnant or breast feeding women, or fertile persons not using contraceptives, were also excluded. The study was approved by the medical ethics committees of both hospitals. All patients gave written informed consent.

### Treatment schedule, dose escalation and dose adjustments

MAG-CPT (provided by Pfizer, Nerviano (Milan), Italy) was administered intravenously in 4-week cycles comprising weekly treatment for three consecutive weeks, followed by 1-week rest. Toxicities were graded according to the CTC of the National Cancer Institute. Dose-limiting toxicities were defined as any of the following events occurring during the first cycle of treatment with MAG-CPT: grade 4 granulocytopenia lasting at least 7 days, febrile neutropenia, neutropenic infection, grade 3 or 4 thrombocytopenia, grade 3 or 4 nonhaematologic toxicity and grade 2 neurotoxicity. Failure to complete the first treatment cycle with three full-weekly doses within a maximum of 6 weeks due to drug-related toxicity was also considered as DLT.

At least three patients were to be treated at the starting dose of 80 mg m^−2^ week^−1^. One patient was treated and observed for 3 weeks from the start of treatment. Thereafter, the second and third patients started with MAG-CPT in the absence of DLTs in the first patient. The latter were observed for at least 3 weeks. If DLT occurred already at the 80 mg m^−2^ week^−1^ (240 mg m^−2^ cycle^−1^) dose level, subsequent treatment would be given at a dose level of 60 mg m^−2^ week^−1^ (180 mg m^−2^ cycle^−1^). If no DLTs or clinically significant toxicity occurred during the first cycle at the starting dose level, further dose escalation would proceed in cohorts of three patients by modified Fibonacci-guided dose increments defined by the investigators on the basis of the clinical and pharmacokinetic results. At subsequent dose levels, three patients were to be enrolled in parallel. At any dose level, if one out of three patients would experience in the first cycle a DLT, the patient cohort had to be enlarged to a total of six patients. If no other DLT or clinically significant toxicity was observed at this dose level, dose escalation would proceed at the next dose increments. The maximum tolerated dose was reached when ⩾2 out of three or ⩾2 out of six patients experience a DLT. Upon identification of the maximum tolerated dose, at least six patients would be treated at the next lower dose level, to better characterise the safety profile at that dose. The next dose level below the maximum tolerated dose would be the starting dose recommended for use in subsequent phase II studies. No within-patient dose escalation was foreseen.

Dose modifications during a cycle were based on worst toxicity attributable to the study drug observed during that given cycle. In case of grade 3/4 granulocytopenia, grade 3/4 thrombocytopenia, febrile neutropenia, neutropenic infection, grade 2–4 neurological toxicity and grade 3/4 other nonhaematologic toxicities, treatment was stopped for the given cycle. Dose adjustment at the start of subsequent cycles was based on the assessment of toxicity during the previous cycle. When patients experienced grade 2–4 granulocytopenia, grade 2–4 thrombocytopenia, grade 1–4 neurotoxicity, or other grade 2–4 nonhaematologic toxicities, treatment was delayed until recovery. If re-treatment had to be held for more than 2 weeks, the patient was removed from therapy.

Prophylactic treatment with antiemetics was not provided at the first dose during the first cycle of treatment. Afterwards, antiemetic prophylactics could be administered based on the judgement of the physician. For diarrhoea loperamide was allowed.

### Treatment evaluation

Patient evaluation on days 1, 8, 15 and 22 of each cycle included complete blood cell counts, liver and renal functions, urinalysis, performance status and toxicity scoring according to the CTC. Tumour measurements were repeated at least every two cycles. Tumour response was assessed according to RECIST criteria (Therasse *et al*, 2000).

### Pharmacokinetics

During the first cycle of treatment, blood samples were collected for pharmacokinetic analysis of MAG-CPT and free CPT. On days 1 and 15, blood samples were drawn just before infusion, immediately after infusion, at 10 min after the end of infusion, and at 1, 4, 8, 12 and 24 h post-infusion. On day 8, one sample was taken prior to infusion, and one at the end of infusion. On days 22 and 29, one blood sample was collected. Pre-cooled heparinised tubes were used for sampling and were immediately placed on ice until being centrifuged at 1200 **g** at 4°C for 10 min. For determination of free CPT levels, 0.25 ml plasma was added to tubes containing 0.75 ml 8.5% phosphoric acid and subsequently mixed. Specimens were stored at −80°C until analysis.

Urine portions were collected during the first cycle on day 1, before the first dose, and post-dose with timed collections between 0–8 and 8–24 h after infusion. Subsequently, urine samples were taken weekly until the start of the second cycle. An aliquot of each urine portion was stored at −20°C until analysis.

A high-performance liquid chromatographic (HPLC) method with fluorescence detection was used for determination of CPT in plasma and urine (Fraier *et al*, 2000). Total CPT levels were determined after hydrolysis of MAG-CPT. Free CPT was extracted from acidified plasma before determination. Bound CPT was calculated by the difference of total minus free CPT. In urine, only total CPT levels were measured. The lower limit of quantification in plasma was 10 ng ml^−1^ for total CPT and 1 ng ml^−1^ for free CPT. In urine, the lower limit of quantification for total CPT was 50 ng ml^−1^.

Pharmacokinetic calculations for carrier-bound and free CPT were performed using both a noncompartmental and compartmental approach. Noncompartmental analysis was executed with the WinNonLin package (version 2.1, Scientific Consulting Inc.). Compartmental analysis was carried out with the MWPharm (version 3.5, Mediware, Groningen, The Netherlands) and ADAPT II (version 4.0, USC, Los Angeles, USA) packages. Curve stripping and subsequent curve fitting was performed in the KINSTRIP and KINFIT programs of the MWPharm package. These data were subsequently used in a multi-compartment analysis in ADAPT II, in which the combined data of carrier bound and free CPT in plasma and the amount CPT excreted in urine were analysed. Variance for the observations was assumed to be proportional to the measured values and set at 10%.

The percentage of the administered dose recovered in urine over the first 24 h was calculated as the amount excreted in urine divided by the total administered dose. The creatinine clearance before treatment was determined according to the Cockroft and Gault formula (Cockcroft and Gault, 1976).

## RESULTS

### Patient characteristics and treatment

Between August 2000 and January 2001, nine patients (four males and five females) were included in the study. Patient characteristics are listed in Table 1

**Table 1:** Patient characteristics

Patients entered	9
Male/female	4/5
Median age, years (range)	55 (26–74)
	
*Performance status*	
0	3
1	6
	
*Malignancy*	
Melanoma	1
Non-small-cell lung cancer	4
Sarcoma	3
Renal cell carcinoma	1
	
*Prior therapy*	
Chemotherapy	8
Radiotherapy	2
Immunotherapy	1
Hormonal therapy	1

. Patients received a total of 20 cycles, with a median of two (range 1–4) cycles per patient.

### Toxicity

Initially, three patients were enrolled at a dose level of 80 mg m^−2^ week^−1^. No DLTs occurred during the first cycle of treatment. Subsequently, three patients were enrolled in parallel at the next dose level of 120 mg m^−2^ week^−1^. As the trial continued, two of the three enrolled patients at the first dose level developed chemical cystitis, inducing cumulative bladder toxicity (Table 2

**Table 2:** Bladder toxicity per individual patient per cycle according to CTC

	**CTC grade dysuria**				**CTC grade haematuria**			
	**Cycle**				**Cycle**			
	**1**	**2**	**3**	**4**	**1**	**2**	**3**	**4**
*Cohort 1 (80 mg m^−2^)*								
Patient 1	0	0	0	0	0	0	0	0
Patient 2	0	1	—	—	0	2	—	—
Patient 3	0	2	3	1	1	2	3	1
								
Patient 7	0	0	—	—	0	0	—	—
Patient 8	0	0	—	—	1	1	—	—
Patient 9	0	—	—	—	0	—	—	—
								
*Cohort 2 (120 mg m^−2^)*								
Patient 4	0	0	0	—	0	0	0	—
Patient 5	0	—	—	—	1	—	—	—
Patient 6	1	—	—	—	0	—	—	—

0=no toxicity, 1–4=toxicity grade according to CTC, —=treatment discontinued.

). One patient at the first dose level developed bladder toxicity during the second cycle (grade 1 dysuria and grade 2 haematuria). He was hospitalised and treatment with MAG-CPT was discontinued. In another patient at the first dose level, bladder toxicity worsened from grade 1 haematuria during the first cycle to grade 2 dysuria and haematuria during the second cycle, and to grade 3 dysuria and haematuria during the third cycle. During the fourth cycle, the dose of MAG-CPT was reduced to 60 mg m^−2^ week^−1^. Subsequently, treatment in this patient was stopped because of progressive disease. Two out of three patients at the second dose level experienced grade 1 bladder toxicity during their first cycle. Treatment of both patients was discontinued after the first cycle for progressive disease (Table 2). Owing to the observed cumulative bladder toxicity, it was decided to enlarge the first cohort (80 mg m^−2^ week^−1^) to a total of six patients. Although only grade 1 bladder toxicity occurred in the three additional patients, bladder toxicity in the second and third patients of the first dose level was considered as DLT. Subsequently, it was decided to stop the trial. De-escalation to a lower dose level was not performed, because this would not result in a higher maximum tolerated dose compared to the previous phase I trial in which a single dose of 240 mg m^−2^ (in a 4-week schedule) was the maximum tolerated dose (De Bono *et al*, 2000).

Haematologic toxicity was mild (Table 3

**Table 3:** Worst haematologic CTC toxicity grade per patient

	**80 mg m^−2^ (no. of patients)**	**120 mg m^−2^ (no. of patients)**
*Anaemia*		
1	2	0
2	2	2
		
*Thrombocytopenia*		
1	1	1
2	0	0
		
*Leukopenia*		
1	2	1
2	0	0
		
*Granulocytopenia*		
1	2	0
2	0	0

). Six patients experienced grade 1 or 2 anaemia. Grade 1 thrombocytopenia, grade 1 leucopenia, and grade 1 granulocytopenia were observed in two, three, and two patients, respectively. No febrile neutropenia was observed, and red blood cell or platelet transfusions were not required.

With the exception of bladder toxicity, nonhaematologic toxicity is listed in Table 4

**Table 4:** Worst nonhaematologic CTC toxicity grade per patient^a^

	**80 mg m^−2^ (No. of patients)**	**120 mg m^−2^ (No. of patients)**
*Bilirubin*		
1	1	0
2	0	0
		
*ASAT*		
1	0	0
2	0	1
		
*ALAT*		
1	0	0
2	0	0
3/4	0	1
		
*Creatinine*		
1	3	1
2	0	0
		
*Mucositis*		
1	3	0
2	0	0
		
*Nausea*		
1	3	3
2	1	0
		
*Vomiting*		
1	0	0
2	1	0
		
*Diarrhoea*		
1	0	1
2	0	0
		
*Fever*		
1	0	0
2	1	0
		
*Infections*		
1	0	0
2	1	0
		
*Skin reactions*		
1	1	0
2	0	0
		
*Anorexia*		
1	2	0
2	0	0
		
*Fatigue*		
1	1	0
2	2	1

a Except bladder toxicity.

. Elevation of serum transaminases was seen in one patient with non-small-cell lung cancer and progression of liver metastases after one cycle. Other toxicities were grade 1 elevation of serum bilirubin and creatinine, grade 1 mucositis, nausea, diarrhoea, skin reaction (rash), anorexia and fatigue. A few patients experienced grade 2 nausea, vomiting, fever, infection, or fatigue.

Overall, chemical cystitis induced grade 1–3 dysuria and/or grade 1–3 haematuria in five out of the nine (56%) patients included in this trial. Dose-limiting toxicity was cumulative bladder toxicity, and 80 mg m^−2^ week^−1^ was considered to be the maximum tolerated dose. The reason for treatment discontinuation was progressive disease in eight patients, and bladder toxicity in one patient.

### Tumour response

No objective tumour responses were seen in six evaluable patients. In two patients the best overall response was stable disease.

### Pharmacokinetics

**Figure 1:**
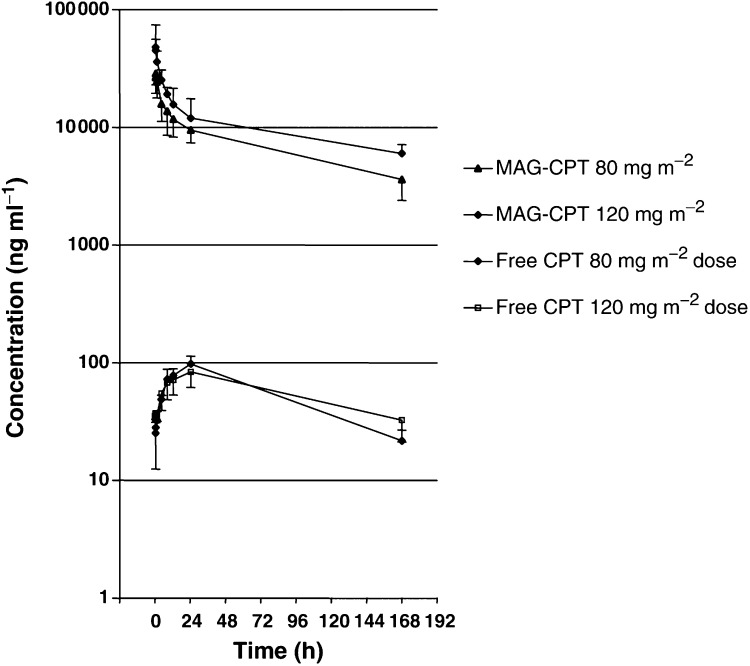
Concentration *vs* time curve for MAG-CPT and free CPT in plasma; values (mean±s.d.).

MAG-CPT plasma data followed a bi- or triexponential elimination pattern in all patients. Simulation of CPT release in the first 24 h after infusion in a two- or three-compartment model with elimination from the first compartment suggested that only 14–20% of the administered dose (expressed as CPT equivalents) had been eliminated after 24 h. Since 26–92% of the dose was recovered in urine after 24 h, this model seems incorrect. Modelling of CPT data in a one-compartment model, while considering MAG-CPT as a mixture of two or three substances each with a different elimination rate, revealed that 60–100% of CPT might have been eliminated after 24 h. One-compartment modelling data also better predicted the measured data (Figure 1

). The main pharmacokinetic parameters of carrier-bound CPT and free CPT in plasma at the first week of treatment are shown in Table 5

**Table 5:** Main pharmacokinetic parameters^a^

	**Carrier-bound CPT (plasma)**	**Free CPT (plasma)**
*80 mg m*^−2^*(n=6)*		
C_end, inf_ (*μ*g ml^−1^)	36.6±13.5	
C_max_ (ng ml^−1^)		102.2±14.3
T_max_ (h)		33.0±10.6
AUC_0-168 h_(mg h l^−1^)	1540±449	10.3±1.2
*V*_1_ (l)	4.6±1.4	
*V*_ss_(l)	15.6±11.0	
*T*_1/2,*α*_ (h)	2.5±1.3	
*T*_1/2,*β*_ (h)	104.5±24.1^b^	42.9±16.9
		
*120 mg/m*^*2*^ *(n=3)*		
*C*_end, inf_ (*μ*g ml^−1^)	23.4±1.3	
*C*_max_ (ng ml^−1^)		85.5±22.6
*T*_max_ (h)		32.2±0.9
AUC_0–168 h_(mg h l^−1^)	1226±483	10.0±2.7
*V*_1_ (l)	4.7±1.9	
*V*_ss_(l)	12.5±4.9	
*T*_1/2,*α*_ (h)	2.4±1.1	
*T*_1/2,*β*_ (h)	100.0±19.8	84.8±21.6

a Values (mean±s.d.) were obtained from the first week of treatment.

b *n*=5, in one patient carrier-bound CPT was not evaluable.

*C*_end, inf_=concentration at the end of infusion, *C*_max_=maximum plasma concentration, *T*_max_=time corresponding to the maximum plasma concentration, AUC_0–168_: area under the plasma concentration–time curve between 0 and 168 h, *V*_1_=initial volume of distribution, *V*_ss_=volume of distribution at steady state, *T*_1/2,*α*_=initial half-life according to one-compartment modelling, *T*_1/2,*β*_=terminal half-life according to one-compartment modelling.

. The fact that the *C*_max_ and AUC_0–168 h_ of free CPT, in patients treated with 80 *vs* 120 mg m^−2^, appeared similar might be explained by the slow release rate of MAG-CPT. Possibly, the period of sampling (between 0 and 24 h after infusion) was too short to determine the true *C*_max_ of free CPT. Urinary excretion during the first 24 h after infusion expressed as a percentage of the total dose administered, as well as the calculated creatinine clearance before treatment, is shown in Table 6

**Table 6:** Urinary excretion of CPT and creatinine clearance

	**Urinary excretion^a^ (% of the dose)**	**Creatinine clearance^b^ (before treatment, ml min^−1^)**
*Cohort 1 (80 mg m*^−2^)		
Patient 1	34.1	60.1
Patient 2	56.2	90.3
Patient 3	91.8	78.2
		
Patient 7	60.3	48.5
Patient 8	52.6	69.3
Patient 9	55.4	45.1
		
Mean±s.d.	58.4±18.8	65.3±17.5
		
*Cohort 2 (120 mg m*^−2^)		
Patient 4	26.4	82.8
Patient 5	38.4	93.7
Patient 6	40.5	76.9
		
Mean±s.d.	35.1±7.6	84.5±8.5

a Values obtained from the first 24 h of treatment (cycle 1).

b Values calculated by Cockroft and Gault formula.

**Figure 2:**
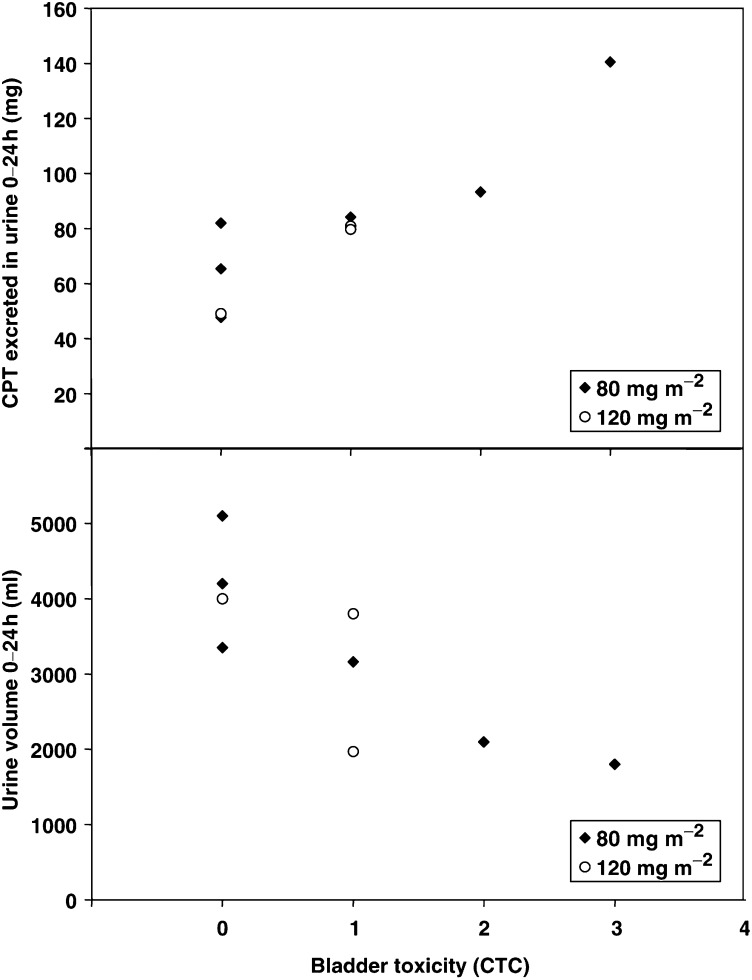
Relation between bladder toxicity and urinary excretion of CPT, and relation between bladder toxicity and urine production between 0 and 24 h after infusion.

. We observed a high variability in urinary excretion between subjects; after 24 h, between 26 and 92% of the total dose was excreted in patients treated with 80 or 120 mg m^−2^ MAG-CPT. The calculated creatinine clearance before treatment was not related to the variability in urinary excretion. Figure 2

shows the relation between the worst bladder toxicity (dysuria or haematuria) observed in individual patients and the total amount of CPT excreted during 24 h after infusion (Spearman's rho 0.82, *P*<0.01). The relation between the worst bladder toxicity and the urine production between 0 and 24 h after infusion is also shown in Figure 2 (Spearman's rho −0.84, *P*<0.01). Figure 2 illustrates that bladder toxicity is associated with local CPT exposure.

## DISCUSSION

In this phase I study, MAG-CPT was administered weekly for 3 weeks in 4-week cycles in adult patients with solid tumours. Dose escalation was already stopped when two patients at the starting dose level developed a disabling haemorrhagic cystitis during the second cycle. Other haematologic or nonhaematologic toxicity was relatively mild. Dose-limiting toxicity was cumulative bladder toxicity, and 80 mg m^−2^ week^−1^ was considered to be the maximum tolerated dose. De-escalation to a lower dose level (60 mg m^−2^ week^−1^) was not performed, because at this dose level a higher maximum tolerated dose could not be reached compared to the previous phase I trial in which a single dose of 240 mg m^−2^ (in a 4-week schedule) was the maximum tolerated dose (De Bono *et al*, 2000). Schoemaker *et al* (2002) showed that, also with MAG-CPT infusion over 3 consecutive days, every 4 weeks the cumulative bladder toxicity was dose-limiting. Dysuria and haematuria, generally developing during the first or second cycle, were noted in patients treated at 68 mg m^−2^ day^−1^ and higher dose levels. As in the previous trials, no objective tumour responses were observed in this trial. Without bladder protection, this weekly regimen of MAG-CPT is not a suitable regimen for treatment of patients with solid tumours.

The action of CPT is most evident in the S-phase of the cell cycle; therefore, prolonged inhibition of topoisomerase I was postulated to be an important parameter in causing cytotoxicity (Hertzberg *et al*, 1989; Del Bino *et al*, 1991; Kingsbury *et al*, 1991; Caiolfa *et al*, 2000). The relation between bladder toxicity and the dose excreted in 24 h suggests that bladder toxicity may be explained by interindividual differences in pharmacokinetics. Accumulation of both carrier-bound and free CPT during subsequent cycles could not be assessed properly, because pharmacokinetics were only performed during the first cycle of treatment. However, clinical data did not demonstrate a reduction in bladder toxicity in the weekly administration schedule compared to the schedule with administration over 3 consecutive days, suggesting that with weekly MAG-CPT treatment accumulation in plasma still plays a role in inducing bladder toxicity. Alkalisation of urine by treatment with sodium bicarbonate might be a possible solution for MAG-CPT-induced bladder toxicity. However, due to the relatively long terminal half-life of MAG-CPT (about 100 h) and the cumulative bladder toxicity after 4 weeks, this treatment was not considered a suitable option. Whether the uroprotective drug mesna has any effect on bladder toxicity induced by MAG-CPT is currently unknown. As bladder toxicity is associated with local CPT exposure, and an inverse relation between urine volume and bladder toxicity was observed in this trial, hyperhydration during MAG-CPT treatment might be useful to reduce bladder toxicity.

Large and variable amounts of CPT are excreted in urine within the first 24 h after infusion. This might be largely due to the linkage of CPT to the water-soluble MAG polymer, since CPT itself is predominantly excreted into bile after intravenous administration (Ahmed *et al*, 1996). The total urinary excretion of the water-soluble CPT analogues irinotecan and topotecan is about 30 and 40%, respectively (Herben *et al*, 1996; Sparreboom *et al*, 1998). The large amount of CPT excreted in urine after administration of MAG-CPT cannot be explained in a multi-compartment pharmacokinetics analysis when only free CPT is supposed to be renally excreted. The plasma levels of free CPT remain relatively low during this period. The polyexponential elimination of MAG-CPT suggests extensive distribution, but it is unlikely that the MAG-CPT macromolecule distributes extensively throughout the body. Therefore, we considered a classical two- or three-compartment model with elimination from the first compartment physiologically incorrect. The pharmacokinetic profile of MAG-CPT may be the result of hybrid release characteristics of CPT from the macromolecule complex. If CPT becomes available with different release rates from the macromolecule complex, rapidly released CPT can account for the large amounts of free CPT found in urine within 24 h after infusion, while more tightly bound CPT results in sustained delivery of CPT from the complex. However, *in vitro* hydrolysis in human plasma showed a free CPT recovery of less than 10% after 24 h of incubation at 37°C. Moreover, in line with the findings of Schoemaker *et al* (2002), we observed equal elimination halve-lives for bound and free CPT, indicating that the kinetics of free CPT are dependent on the release rate from its carrier. Thus, not plasma, but certain tissue sites might be responsible for the rapid release of free CPT into urine. Since renal excretion is the main route of elimination, the kidney may be a significant site of hydrolysis of CPT from its polymeric carrier. Renal absorption and subsequent catabolism has previously been shown to occur for drug–protein conjugates in proximal tubular cells of the kidneys (Franssen *et al*, 1992). This might also be the fate of drug–MAG polymers. Rapid elimination of CPT into urine immediately after hydrolysis might explain why the free CPT plasma levels remain relatively low.

In spite of the special pharmacokinetic properties of MAG-CPT, a favourable impact on pharmacodynamics was not shown. An interesting approach to investigate CPT accumulation in human tumour tissue was recently published by Sarapa *et al*. Normal and tumour tissue uptake of MAG-CPT was assessed in patients undergoing elective surgery for colorectal carcinoma (Sarapa *et al*, 2003). Patients received a single dose of 60 mg m^−2^ of MAG-CPT either 24 h, 3 days, or 7 days prior to surgery. They demonstrated delivery of CPT to the target tumour tissue and found the equilibrium between plasma and tumour tissue concentrations of released CPT being established within 24 h after dosing. No evidence for selective delivery or retention of MAG-CPT or preferential release of free CPT in tumour tissue was found (Sarapa *et al*, 2003).

Other phase I trials with polymeric drug conjugates based on *N*-(2-hydroxypropyl)methacrylamide copolymers bound to doxorubicin and paclitaxel have been performed (Muggia, 1999; Vasey *et al*, 1999; Seymour *et al*, 2002). Vasey *et al* (1999) showed that PK1 (doxorubicin covalently bound to *N*-(2-hydroxypropyl)methacrylamide) decreases doxorubicin DLTs, maintained antitumour efficacy, and demonstrated no polymer-related toxicity. A phase I study of PNU-166945 (a polymer-conjugated prodrug of paclitaxel) was discontinued prematurely, mainly due to severe neurotoxicity in additional rat studies (Meerum Terwogt *et al*, 2001).

Drug delivery based on polymers is an interesting approach in the treatment of solid tumours. Binding of antitumour agents to hydrophilic polymers can improve the solubility of poorly water-soluble drugs and induce tumour targeting. However, polymeric drug conjugates have an altered pharmacokinetic and toxicity profile. Pharmacokinetics in this study revealed a direct association between bladder toxicity and local CPT exposure. Due to disabling bladder toxicity, weekly MAG-CPT is not a suitable regimen.

## References

[bib1] Ahmed AE, Jacob S, Giovanella BC, Kozielski AJ, Stehlin Jr JS, Liehr JG (1996) Influence of route of administration on [^3^H]-camptothecin distribution and tumor uptake in CASE-bearing nude mice: whole-body autoradiographic studies. Cancer Chemother Pharmacol 39: 122–130 8995509 10.1007/s002800050547

[bib2] Caiolfa VR, Zamai M, Fiorino A, Frigerio E, Pellizzoni C, D'Argy R, Ghiglieri A, Castelli MG, Farao M, Pesenti E, Gigli M, Angelucci F, Suarato A (2000) Polymer-bound camptothecin: initial biodistribution and antitumour activity studies. J Control Release 65: 105–119 10699275 10.1016/s0168-3659(99)00243-6

[bib3] Cockcroft DW, Gault MH (1976) Prediction of creatinine clearance from serum creatinine. Nephron 16: 31–41 1244564 10.1159/000180580

[bib4] De Bono JS, Bissett D, Twelves C, Main M, Muirhead F, Robson L, Fraier D, Magne MI, Porro M, Speed W, Cassidy J (2000) Phase I pharmacokinetic (PK) study of MAG-CPT (PNU 166148), a polymeric derivative of camptothecin (CPT). Proc Am Soc Clin Oncol 19: 198 10.1038/sj.bjc.6601922 PMC2364737 15187995

[bib5] Del Bino G, Lassota P, Darzynkiewicz Z (1991) The S-phase cytotoxicity of camptothecin. Exp Cell Res 193: 27–35 1995300 10.1016/0014-4827(91)90534-2

[bib6] Duncan R (1999) Polymer conjugates for tumour targeting and intracytoplasmic delivery. The EPR effect as a common gateway? Pharm Sci Technol Today 2: 441–449 10542390 10.1016/s1461-5347(99)00211-4

[bib7] Fraier D, Frigerio E, Brianceschi G, Casati M, Benecchi A, James C (2000) Determination of MAG-camptothecin, a new polymer-bound camptothecin derivative, and free camptothecin in dog plasma by HPLC with fluorimetric detection. J Pharm Biomed Anal 22: 505–514 10766368 10.1016/s0731-7085(99)00315-5

[bib8] Franssen EJ, Koiter J, Kuipers CA, Bruins AP, Moolenaar F, de Zeeuw D, Kruizinga WH, Kellogg RM, Meijer DK (1992) Low molecular weight proteins as carriers for renal drug targeting. Preparation of drug–protein conjugates and drug-spacer derivatives and their catabolism in renal cortex homogenates and lysosomal lysates. J Med Chem 35: 1246–1259 1560438 10.1021/jm00085a012

[bib9] Gottlieb JA, Guarino AM, Call JB, Oliverio VT, Block JB (1970) Preliminary pharmacologic and clinical evaluation of camptothecin sodium (NSC-100880). Cancer Chemother Rep 54: 461–470 4946015

[bib10] Herben VM, Bokkel Huinink WW, Beijnen JH (1996) Clinical pharmacokinetics of topotecan. Clin Pharmacokinet 31: 85–102 8853931 10.2165/00003088-199631020-00001

[bib11] Hertzberg RP, Caranfa MJ, Hecht SM (1989) On the mechanism of topoisomerase I inhibition by camptothecin: evidence for binding to an enzyme–DNA complex. Biochemistry 28: 4629–4638 2548584 10.1021/bi00437a018

[bib12] Kim CK, Lim SJ (2002) Recent progress in drug delivery systems for anticancer agents. Arch Pharm Res 25: 229–239 12135091 10.1007/BF02976620

[bib13] Kingsbury WD, Boehm JC, Jakas DR, Holden KG, Hecht SM, Gallagher G, Caranfa MJ, McCabe FL, Faucette LF, Johnson RK (1991) Synthesis of water-soluble (aminoalkyl)camptothecin analogues: inhibition of topoisomerase I and antitumor activity. J Med Chem 34: 98–107 1846923 10.1021/jm00105a017

[bib14] Maeda H, Matsumura Y (1989) Tumoritropic and lymphotropic principles of macromolecular drugs. Crit Rev Ther Drug Carrier Syst 6: 193–210 2692843

[bib15] Maeda H, Wu J, Sawa T, Matsumura Y, Hori K (2000) Tumor vascular permeability and the EPR effect in macromolecular therapeutics: a review. J Control Release 65: 271–284 10699287 10.1016/s0168-3659(99)00248-5

[bib16] Meerum Terwogt JM, Ten Bokkel Huinink WW, Schellens JH, Schot M, Mandjes IA, Zurlo MG, Rocchetti M, Rosing H, Koopman FJ, Beijnen JH (2001) Phase I clinical and pharmacokinetic study of PNU166945, a novel water-soluble polymer-conjugated prodrug of paclitaxel. Anticancer Drugs 12: 315–323 11335787 10.1097/00001813-200104000-00003

[bib17] Muggia FM (1999) Doxorubicin–polymer conjugates: further demonstration of the concept of enhanced permeability and retention. Clin Cancer Res 5: 7–8 9918196

[bib18] Muggia FM, Creaven PJ, Hansen HH, Cohen MH, Selawry OS (1972) Phase I clinical trial of weekly and daily treatment with camptothecin (NSC-100880): correlation with preclinical studies. Cancer Chemother Rep 56: 515–521 5081595

[bib19] Sarapa N, Britto MR, Speed W, Jannuzzo M, Breda M, James CA, Porro M, Rocchetti M, Wanders A, Mahteme H, Nygren P (2003) Assessment of normal and tumor tissue uptake of MAG-CPT, a polymer-bound prodrug of camptothecin, in patients undergoing elective surgery for colorectal carcinoma. Cancer Chemother Pharmacol 52: 424–430 12904897 10.1007/s00280-003-0685-x

[bib20] Schoemaker NE, Van Kesteren C, Rosing H, Jansen S, Swart M, Lieverst J, Fraier D, Breda M, Pellizzoni C, Spinelli R, Porro M, Beijnen JH, Schellens JH, Ten Bokkel Huinink WW (2002) A phase I and pharmacokinetic study of MAG-CPT, a water-soluble polymer conjugate of camptothecin. Br J Cancer 87: 608–614 12237769 10.1038/sj.bjc.6600516 PMC2364251

[bib21] Seymour LW, Ferry DR, Anderson D, Hesslewood S, Julyan PJ, Poyner R, Doran J, Young AM, Burtles S, Kerr DJ (2002) Hepatic drug targeting: phase I evaluation of polymer-bound doxorubicin. J Clin Oncol 20: 1668–1676 11896118 10.1200/JCO.2002.20.6.1668

[bib22] Sparreboom A, de Jonge MJ, de Bruijn P, Brouwer E, Nooter K, Loos WJ, van Alphen RJ, Mathijssen RH, Stoter G, Verweij J (1998) Irinotecan (CPT-11) metabolism and disposition in cancer patients. Clin Cancer Res 4: 2747–2754 9829738

[bib23] Therasse P, Arbuck SG, Eisenhauer EA, Wanders J, Kaplan RS, Rubinstein L, Verweij J, Van Glabbeke M, Van Oosterom AT, Christian MC, Gwyther SG (2000) New guidelines to evaluate the response to treatment in solid tumors. European Organization for Research and Treatment of Cancer, National Cancer Institute of the United States, National Cancer Institute of Canada. J Natl Cancer Inst 92: 205–216 10655437 10.1093/jnci/92.3.205

[bib24] Vasey PA, Kaye SB, Morrison R, Twelves C, Wilson P, Duncan R, Thomson AH, Murray LS, Hilditch TE, Murray T, Burtles S, Fraier D, Frigerio E, Cassidy J (1999) Phase I clinical and pharmacokinetic study of PK1 [*N*-(2-hydroxypropyl)methacrylamide copolymer doxorubicin]: first member of a new class of chemotherapeutic agents–drug–polymer conjugates. Cancer Research Campaign Phase I/II Committee. Clin Cancer Res 5: 83–94 9918206

